# Estimation of the pooled mean blood lead levels of Indian children: Evidence from systematic review and meta-analysis

**DOI:** 10.1016/j.toxrep.2025.101975

**Published:** 2025-02-25

**Authors:** Kuldip Upadhyay, Rakesh Balachandar, Bhavani Shankara Bagepally, Kalahasthi Ravibabu, Venugopal Dhananjayan, Nagaraju Raju, Geetika Yadav, Beerappa Ravichandran, Santasabuj Das

**Affiliations:** a ICMR-National Institute of Occupational Health, Ahmedabad, India; b ICMR-NIOH-Regional Occupational Health Centre (S), Bengaluru, India; c ICMR-National Institute of Epidemiology, Chennai, India; d Indian Council of Medical Research, New Delhi, India

**Keywords:** Lead poisoning, High risk group, Unknown exposure group, Time trend analysis, Environmental intervention

## Abstract

A recent systematic review reported very high pooled estimates of blood lead levels (BLLs) for Indian children. Current study aimed at systematically pooling the BLLs of Indian children (aged ≤ 14 years). Further, explore the time trend of BLLs with respect to implementing the ban on the use of Pb-petrol (i.e.2000) and a decade later (2010). Observational studies documenting the BLL in Indian children (aged ≤ 14 years) from PubMed-Medline, Scopus, and Embase digital databases from inception to August 2024 were systematically reviewed. Detailed protocol is available at PROSPERO (ID: CRD42022382835). Pooled mean BLL was estimated using the random-effects model and conventional-*I*^*2*^ statistics to assess the heterogeneity, while the Newcastle Ottawa Scale for bias assessment. Sub-group, sensitivity, and meta-regression analyses were performed where data permitted. Observations from 65 reports (51 original studies) revealed pooled BLL of 10.4 (95 % CI: 9.55–11.2) µg/dL with a trend of gradual reduction during the last 3 decades. Subgroup analysis revealed the high risk (with known Pb exposure) children had BLL of 14.3 (12.3–16.2) µg/dL, while that of the low risk (no known Pb exposure) is 8.71 (7.71–9.71) µg/dL. Only the low risk group exhibited a time trend of a gradual reduction in BLL. Notably, the review observed high heterogeneity. A progressive decline in Pb burden with respect to the national ban on leaded petrol was observed. However, present observations emphasize remedial actions toward non-occupational Pb exposure particularly among high risk Pb group, such as periodic BLL surveys.

## Highlights

• Pooled data indicates Indian children's mean BLL is above the reference level.
• BLLs have gradually declined following the ban on leaded petrol in India.
• Higher BLLs observed in children with known/high Pb exposure risk versus low risk.
• Currently available evidences are predominantly low quality and carried high heterogeneity.
• National programs are effective and required on long run to reduce Pb pollution

## Graphical Abstract

**Figure:**
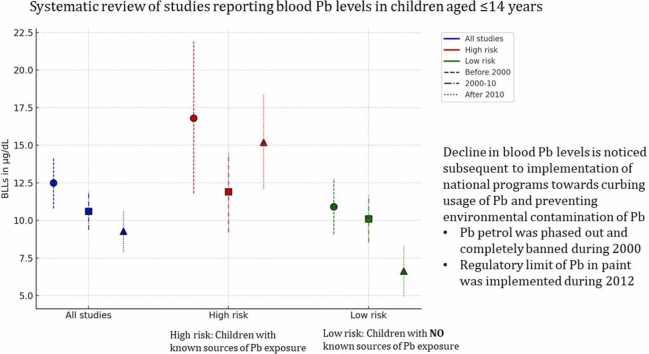

## 1 Introduction

Lead (Pb) exposure is a significant public health concern as it accounts for 0.9 million deaths and 21.7 million disability-adjusted life years (DALY) globally due to long-term Pb exposure [1]. Children are particularly vulnerable to chronic Pb exposure, and WHO estimated 30 % of idiopathic developmental intellectual disabilities attributable to chronic Pb exposure [2]. As Pb was detected in > 80 % of the general population (with no known Pb exposure), despite lacking any known physiologic role, the Centre for Disease Control and Prevention (CDC, USA) set blood lead level (BLL) < 3.5 µg/dL as the “reference level”. Hence CDC recommended individuals with BLL > reference levels should be investigated for the potential sources of Pb exposure to prevent further exposure and reduce the Pb burden [3].

Globally, tetraethyl Pb usage in gasoline was the major source of airborne Pb pollution and Pb exposure [4], [5], [6], [7], [8], [9], [10]. Leaded petrol was phased out in India in 1996 and completely banned in 2000 resulting in a gradual reduction in airborne Pb exposure reflected as declining BLL. Despite the initial decline following the ban on leaded petrol, the BLL continued to persist even after a decade [11], [12], [13], [14].

Persistently raised BLL after a ban on leaded petrol was suspected due to environmental exposure, in view of the residences proximal to Pb-based industries [11], [12], [13]. Further, traces of Pb detected in traditional medicinal preparations (ayurvedic), leaded paint, cosmetics (kohl/surma), and certain food products are proposed sources of Pb exposure among Indian children [14], [15], [16], [17], [18], [19]. National stakeholders periodically implement interventions toward reducing the Pb burden. One such intervention included the prohibition of the manufacture, trade, import, and export of household and decorative paints with Pb contents of more than 90 parts per million (ppm) [20].

A recent meta-analysis of BLL measured in Indian children during 2010–2018 reported an average four (4) IQ point loss attributable to Pb exposure. The study reviewed seventeen reports and observed a mean BLL of 6.86 µg/dL [21]. Recently, a joint report by UNICEF and Pure Earth (a non-government non-profit international organization) revealed that one in every third Indian child had BLL > 5 µg/dL [3]. The data reported by these international organizations was verified by the Government of India (NITI Aayog) in association with the Council of Scientific & Industrial Research (CSIR), [22]. The report lacked the effects of periodic national interventions toward reducing the Pb burden and failed to identify the risk (Pb exposure) groups for specific interventions. The current study aimed at systematically pooling the BLLs of Indian children available from the current literature. Conventionally, engaging children ≤ 14 years in any form of labor are prohibited as defined by Child Labor Prohibition and Regulation Act - 1986 [23]. Therefore, current study reviewed the studies, those involving children aged ≤ 14 years. Further, we explore the time trend of BLLs with respect to ban on use of leaded petrol (i.e. in the year 2000) and strict imposition of the ban a decade later (i.e. 2010).

## 2 Method

The present systematic review estimated the pooled BLLs of Indian children aged ≤ 14 years, from the available literature. Scopus, Embase, and PubMed are systematically searched for original studies available from their inception to August 1, 2024, reporting BLLs among Indian children aged ≤ 14 years (supplement Table 1 search terms). All Indian studies reporting BLL of children aged ≤ 14 years irrespective sex and region of residency are included. Reviews, commentaries, and methodology publications are excluded. The study complied with “Preferred Reporting Items of Systematic Review and Meta-analysis - (PRISMA)” guidelines and the protocol is made available at Prospero (CRD42022382835) [24].

**Table 1:** Description of individual studies and the participants.

Citation	Study site and year of participant recruitment	Sample size & % of male participants	Age (in years) of the participants reported as Mean (SD) / range	Method of lead estimation, and description of QC check	Risk of exposure and prevalence of high BLL	Site of participant recruitment
Lu et al. [38]	Bihar; 2022	n = 697; 55 % of male participants	1. – 5	Anodic Stripping Voltammetry and QC tested	Risk of exposure is low	Community
Sahu et al. [39]	Ahmedabad; 2021	n = 90	< 1	Flameless Atomic Absorption Spectrophotometry	Risk of exposure is low	Community
Kumar et al. [40]	Multiple Cities; 2020	n = 1143; 49.5 % of male participants	6–11	Inductively Coupled Plasma Optical Emission Spectroscopy	Risk of exposure is low	Community
Abinaya et al. [41]	Tamil Nadu; 2022	n = 83	< 1	Inductively Coupled Plasma Optical Emission Spectroscopy	Risk of exposure is low	Community
Mahdi et al. [42]	Uttar Pradesh; 2022	n = 200	< 1	Inductively Coupled Plasma Optical Emission Spectroscopy	Risk of exposure is low	Community
Brown et al. [43] (A)^#^	Bihar; 2020	n = 67; % of male participants = 58	0.67 – 5.92	Anodic Stripping Voltammetry and QC tested	Risk of exposure is high	Community
Brown et al. [43] (B)^#^	Bihar; 2020	n = 68; % of male participants = 50	0.67 – 5.92	Anodic Stripping Voltammetry and QC tested	Risk of exposure is low	Community
Ramesh et al. [44] (A)^#^	Tamil Nadu; 2015	n = 87; % of male participants = 42.5	2.1–4.6	Graphite Furnace Atomic Absorption Spectrophotometry	Risk of exposure is high	Community
Ramesh et al. [44] (B)^#^	Tamil Nadu; 2015	n = 66; % of male participants = 53	2.1–4.6	Graphite Furnace Atomic Absorption Spectrophotometry	Risk of exposure is low	Community
Rawat et al. [45]	Uttar Pradesh; 2022*	n = 43	4–12	Anodic Stripping Voltammetry and QC tested	Risk of exposure is high	Community
Shekhawat. [46]	Rajasthan; 2021*	n = 167; % of male participants = 48	< 1	Inductively Coupled Plasma Optical Emission Spectroscopy and QC tested	Risk of exposure is low; Prevalence of high BLL is 51 %	Community
Malavika L. et al. [47]	Rajasthan; 2021*	n = 20	9–13	Graphite Furnace Atomic Absorption Spectrophotometry and QC tested	Risk of exposure is low; Prevalence of high BLL is 36 %	Community
Ghosh et al. [28]	Rajasthan; 2021*	n = 82; % of male participants = 46	12 (3)	Graphite Furnace Atomic Absorption Spectrophotometry and QC tested	Risk of exposure is low	Community
Ansari et al. [48]	Bihar; 2018	n = 41; % of male participants = 23	7.7 (2.3)	Anodic Stripping Voltammetry	Risk of exposure is high; Prevalence of high BLL is 91 %	Community
Koshy et al. [49]	Tamil Nadu; 2012	n = 228; % of male participants = 46	1.3	Graphite Furnace Atomic Absorption Spectrophotometry	Risk of exposure is low	Community
Rashid et al. [35]	Jammu & Kashmir; 2019*	n = 25; % of male participants = 59	2–12	Atomic Absorption Spectrophotometry	Risk of exposure is low; Prevalence of high BLL is 28 %	Community
Goel and Chowgule [50] (A)^#^	Maharashtra; 2011	n = 15; % of male participants = 60	3.9 (4.1)	Differential Pulse Anodic Stripping Voltammetry	Risk of exposure is high	Hospital
Goel and Chowgule. [50] (B)^#^	Maharashtra; 2011	n = 14; % of male participants = 50	5.3 (1.9–13)	Differential Pulse Anodic Stripping Voltammetry	Risk of exposure is low	Community
Sehgal et al. [30] (A)^#^	New Delhi; 2019*	n = 60	3–12	Inductively Coupled Plasma Atomic Emission Spectroscopy	Risk of exposure is high	Hospital
Sehgal et al. [30] (B)^#^	New Delhi; 2019*	n = 60	3–12	Inductively Coupled Plasma Atomic Emission Spectroscopy	Risk of exposure is low	Hospital
Santra et al. [51] (A)^#^	West Bengal; 2018	n = 70; % of male participants = 60	1–10	Inductively Coupled Plasma Mass Spectrometry	Risk of exposure is high	Hospital
Santra et al. [51] (B)^#^	West Bengal; 2018	n = 30; % of male participants = 63	1–10	Inductively Coupled Plasma Mass Spectrometry	Risk of exposure is low	Community
Maheshwari et al. [52] (A)^#^	Karnataka; 2014	n = 60	9–12	Anodic Stripping Voltammetry	Risk of exposure is high; Prevalence of high BLL is 16 %	Community
Maheshwari et al. [52] (B)^#^	Karnataka; 2014	n = 60	9–12	Anodic Stripping Voltammetry	Risk of exposure is low	Community
Chaudhary et al. [53]	Uttar Pradesh; 2018	n = 260; % of male participants = 59.6	4.6 (0.9)	Inductively Coupled Plasma Optical Emission Spectroscopy	Risk of exposure is high; Prevalence of high BLL is 44.2 %	Hospital
Pratinidhi et al. [54] (A)^#^	Maharashtra; 2014*	n = 30; % of male participants = 60	3–12	Anodic Stripping Voltammetry	Risk of exposure is high; Prevalence of high BLL is 9 %	Hospital
Pratinidhi et al. [54] (B)^#^	Maharashtra; 2014*	n = 30; % of male participants = 60	3–12	Anodic Stripping Voltammetry	Risk of exposure is low	Community
Kalra et al. [55]	Delhi; 2006	n = 300; % of male participants = 40	8.4 (1.8)	Anodic Stripping Voltammetry and QC tested	Risk of exposure is low; Prevalence of high BLL is 36 %	Community
Goswami [14] (A)^#^	West Bengal; 2013*	n = 69; % of male participants = 29	6.7 (2.9)	Atomic Absorption Spectrophotometry	Risk of exposure is high	Hospital
Goswami [14] (B)^#^	West Bengal; 2013*	n = 24; % of male participants = 29	6.4 (3.2)	Atomic Absorption Spectrophotometry	Risk of exposure is low	Hospital
Patel et al. [56]	Maharashtra; 2001	n = 200	0.4–0.9	Graphite Furnace Atomic Absorption Spectrophotometry	Risk of exposure is high; Prevalence of high BLL is 38.2 %	Community
Palaniappan et al. [57]	Tamil Nadu; 2006	n = 756; % of male participants = 53	3–7	Anodic Stripping Voltammetry and QC tested	Risk of exposure is high; Prevalence of high BLL is 52.5 %	Community
Reddy et al. [58] (A)^#^	Telangana; 2011*	n = 130	12.3 (1.71)	Anodic Stripping Voltammetry	Risk of exposure is high; Prevalence of high BLL is 54.3 %	Community
Reddy et al. [58] (B)^#^	Telangana; 2011*	n = 65	12.2 (1.48)	Anodic Stripping Voltammetry	Risk of exposure is low; Prevalence of high BLL is 54.3 %	Community
Hegde et al. [59]	Rajasthan; 2010*	n = 100; % of male participants = 67	5–13	Atomic Absorption Spectrophotometry	Risk of exposure is high	Community
Choudhari et al. [32] (A)^#^	Rajasthan; 2010*	n = 298; % of male participants = 53.3	9–14	Graphite Furnace Atomic Absorption Spectrophotometry	Risk of exposure is high	Community
Choudhari et al. [32] (B)^#^	Rajasthan; 2010*	n = 154; % of male participants = 50.6	9–14	Graphite Furnace Atomic Absorption Spectrophotometry	Risk of exposure is low	Community
Chaudhary and Sharma [33]	Uttar Pradesh; 2009	n = 100	3–5	QC tested	Risk of exposure is low	Community
Ahamed et al. [15]	Uttar Pradesh; 2006	n = 200; % of male participants = 73.5	3–12	Graphite Furnace Atomic Absorption Spectrophotometry and QC tested	Risk of exposure is low; Prevalence of high BLL is 37 %	Community
Patel and Athawale [60]	Maharashtra; 2001	n = 91	6.13 (3.4)	Flameless Atomic Absorption Spectrophotometry	Risk of exposure is high	Hospital
Patel and Prabhu [60]	Maharashtra; 2009*	n = 205	< 1	Flameless Atomic Absorption Spectrophotometry	Risk of exposure is low	Community
Ahamed et al. [61] (A)^#^	Uttar Pradesh; 2008*	n = 30	7.73 (2.26)	Graphite Furnace Atomic Absorption Spectrophotometry and QC tested	Risk of exposure is high; Prevalence of high BLL is 90 %	Community
Ahamed et al. [61] (B)^#^	Uttar Pradesh; 2008*	n = 60; % of male participants = 72	6.88 (2.59)	Graphite Furnace Atomic Absorption Spectrophotometry and QC tested	Risk of exposure is low	Hospital
Ahamed et al. [62]	Uttar Pradesh; 2007*	n = 75; % of male participants = 50.6	1–7	Graphite Furnace Atomic Absorption Spectrophotometry	Risk of exposure is high	Community
Zimmermann et al. [31]	Karnataka; 2005	n = 134	7.15 (1.5)	Anodic Stripping Voltammetry and QC tested	Risk of exposure is low	Community
Jain and Hu [34]	Maharashtra & Delhi; 1999	n = 1076	0–3	Anodic Stripping Voltammetry	Risk of exposure is low	Community
Nichani et al. [63]	Maharashtra; 2003	n = 754; % of male participants = 53.3	0–12	Anodic Stripping Voltammetry	Risk of exposure is low; Prevalence of high BLL is 33.4 %	Community
Mahajan et al. [64]	Punjab; 2004	n = 160; % of male participants = 62.5	0.3–6	Anodic Stripping Voltammetry and QC tested	Risk of exposure is high	Hospital
Bellinger et al. [11]	Tamil Nadu; 2005*	n = 74	4–14	Anodic Stripping Voltammetry	Risk of exposure is low	Community
Kalra et al., 2003 [65]	Delhi; 1998	n = 190	4–6	Anodic Stripping Voltammetry	Risk of exposure is low; Prevalence of high BLL is 18.4 %	Community
D’Souza et al. [66]	Karnataka; 2002*	n = 27; % of male participants = 26.6	4–12	Anodic Stripping Voltammetry	Risk of exposure is high	Community
Mukherjee et al. [67]	West Bengal; 2002*	n = 17	13	Atomic Absorption Spectrophotometry	Risk of exposure is high	Community
Patel et al. [68]	Maharashtra; 1996	n = 297; % of male participants = 52.8	0.5–6	Flame less Atomic Absorption Spectrophotometry	Risk of exposure is high; Prevalence of high BLL is 67 %	Community
Tripathi et al. [36]	Maharashtra & Telangana; 1998	n = 576	3–6	Differential Pulse Anodic Stripping Voltammetry and QC tested	Risk of exposure is low	Community
Srivastava et al. [69] (A)^#^	Uttar Pradesh; 2001*	n = 24	< 1	Graphite Furnace Atomic Absorption Spectrophotometry	Risk of exposure is high	Hospital
Srivastava et al. [69] (B)^#^	Uttar Pradesh; 2001*	n = 23	< 1	Graphite Furnace Atomic Absorption Spectrophotometry	Risk of exposure is low	Hospital
Raghunath et al. [70]	Maharashtra; 1997	n = 148	< 1	Differential Pulse Anodic Stripping Voltammetry and QC tested	Risk of exposure is low	Community
Kaul [71] (A)^#^	Jammu & Kashmir; 1999*	n = 125	3–5	Anodic Stripping Voltammetry and QC tested	Risk of exposure is low; Prevalence of high BLL is 50.5 %	Community
Kaul [71] (B)^#^	New Delhi; 1999*	n = 46	3–5	Graphite Furnace Atomic Absorption Spectrophotometry	Risk of exposure is high	Hospital
Kumar et al. [72] (A)^#^	UttarPradesh; 1998*	n = 82	1–12	Atomic Absorption Spectrophotometry	Risk of exposure is high	Hospital
Kumar et al. [72] (B)^#^	UttarPradesh; 1998*	n = 28	1–12	Atomic Absorption Spectrophotometry	Risk of exposure is low	Community
Raghunath et al. [73]	Maharashtra; 1997*	n = 19	6–10	Differential Pulse Anodic Stripping Voltammetry and QC tested	Risk of exposure is low	Community
Gogte et al. [74] (A)^#^	Delhi; 1991*	n = 82	0.2–13	Colorimetry	Risk of exposure is low	Hospital
Gogte et al. [74] (B)^#^	Delhi; 1991*	n = 125	1–11	Colorimetry	Risk of exposure is high	Hospital
Khandekar et al. [75]	Maharashtra; 1987*	n = 178	0–12	Differential Pulse Anodic Stripping Voltammetry	Risk of exposure is low	Community
Kaul and Kaul [76]	Jammu & Kashmir; 1986*	n = 112	5.9	Anodic Stripping Voltammetry	Risk of exposure is high	Hospital

^#^ Participants with a low and high risk of Pb exposure are reported separately in these studies described as “A” & “B”

* As studies haven’t clarified the study period, the year of publication is regarded as the study period

The steps such as. screening for eligible articles and extracting relevant data and its management are detailed elsewhere [25], [26]. Briefly, all citations are pooled and the study authors independently screened the title and abstract for their inclusion (RB & KU), using the cloud-based application “Rayyan intelligent systematic review” [27]. Thereafter, the full text of screened articles is reviewed for their final inclusion in the systematic review. Relevant data from the included articles are extracted into the pre-validated data extraction sheet. All conflicts during the independent review were resolved by mutual consensus.

Study details such as title, authors, publication year, period and site of data collection, participant details such as sample size, age, gender, residing location, type of sample collected for BLL estimation, analytical method for estimating BLL as well the quality check (QC) for analytical methods and presence of a nearby known source of Pb exposure, and BLLs are extracted from included studies, wherever available and recorded using Google sheets. In cases where data was unavailable, the respective authors were contacted via email to request the required details. Up to three emails were sent, each spaced two weeks apart. If no response was received after these attempts, the data was considered unavailable. For studies without the description of study period, the year of publication is regarded as the study year. Duplicates are identified and excluded by verifying study details viz. authors, study site, period of participant recruitment, and sample size.

Extracted data included the central tendency in terms of mean and median, and data dispersion in terms of standard deviation (SD), inter quartile range (IQR), 95 % confidence interval (CI), standard error of mean (SEM), and range. Standard conversions are applied to derive mean and SD for uniform reporting, when the data is available in alternate forms [28], [29], [30], [31]. Numerical values are extracted using webPlotDigitizer when relevant data is graphically reported [32]. The grand mean is estimated for studies reporting more than one exposed group [31], [32], [33], [34], [35], [36]. Pooled mean BLL is estimated using the generic inverse variance method by pooling the individual mean and SD values. Random-effects model has employed post-confirmation of high heterogeneity among the studies.

The assessment and interpretation of heterogeneity among the included studies, subgroup analyses, sensitivity and meta-regression are executed in accordance to the standard practices [25], [26]. Additionally, the Galbraith plot and leave-one-out analyses are performed to explore the heterogeneity and identify the outliers. Further, the study investigated the trend of BLLs among Indian children before the implementation of unleaded fuel (i.e. 2000), after the imposition of strict guidelines for reducing community Pb burden (i.e. 2010), and between these 2 events by performing subgroup analysis, cumulative meta-analysis, and bubble plot analysis.

For community-based studies, Indian children with known Pb exposure i.e. residing adjacent to a known source of exposure such as the Pb processing industry, or with household sources like house paint, surma/cosmetics, pica, maternal, and parental/para-occupational exposure considered as high-risk Pb exposure group. While children with no known exposure are regarded as an unknown exposure / low risk / general population group

For hospital-based studies, participants (children ≤ 14 years) recruited at the hospital as cases were treated as known exposure, and considered as a known/high-risk Pb exposure group, while the controls of the same studies with no known exposure were considered an unknown exposure / general population group.

The Newcastle Ottawa quality assessment scale is customized to the current study and employed independently for assessing the risk of bias among the included studies [37] (description available in supplement material). Briefly, the tool is used for rating the risk of bias in the participant selection and assessing the exposure. Individual studies are rated with a star(s) based on the description and clarity of the definition of participants, their representativeness, and ascertainment of exposure.

## 3 Results

The electronic search retrieved 8295 studies. After the removal of duplicates, and screening of abstracts & titles, 148 studies are considered for full-text review. Full-text scrutiny resulted in 51 studies for the final quantitative data synthesis. The number of studies excluded, and the reasons are described in the PRISMA flowchart (Supplement Fig. 1).

**Fig. 1:**
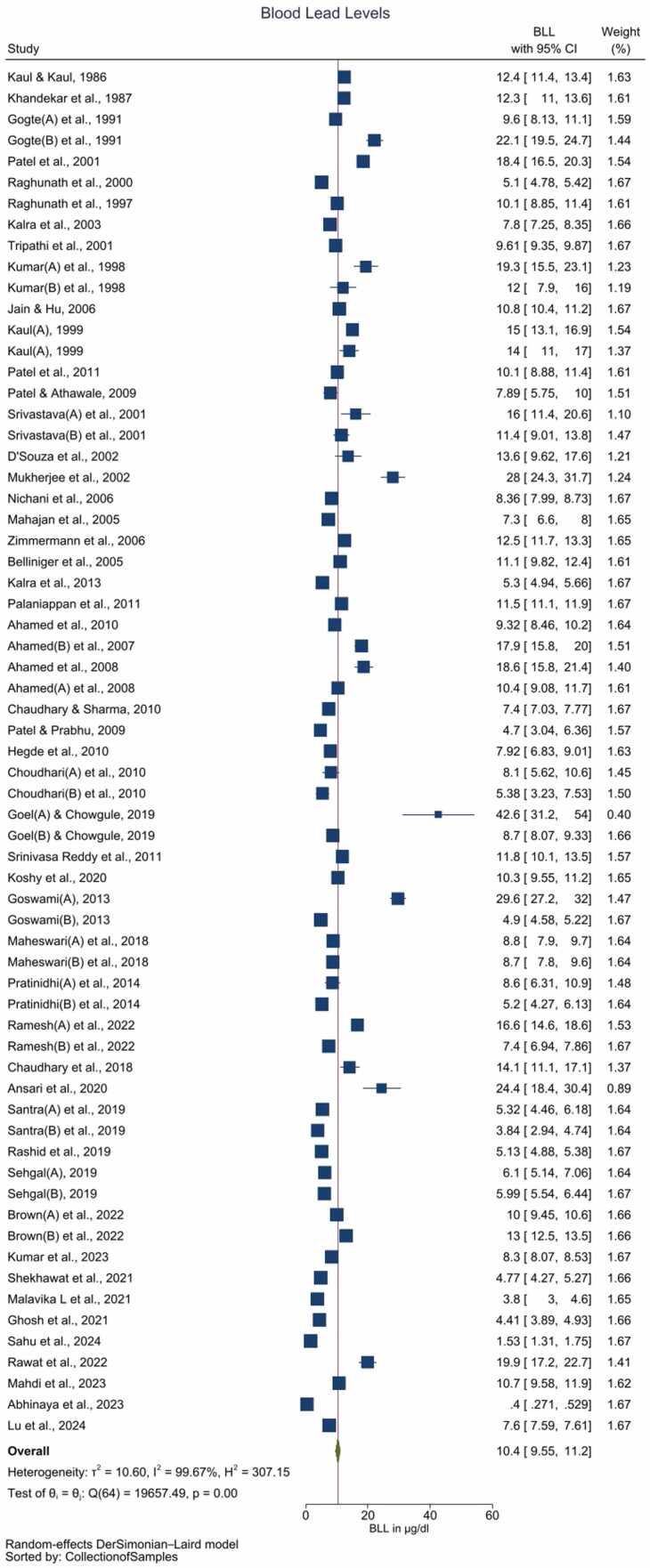
Forest plot for pooled BLLs among Indian children aged ≤ 14 years (Legends / footnotes) Forest plot demonstrates the pooled Pb levels of Indian children is 10.4 (95 % CI: 9.55–11.2) µg/dL with unacceptably high heterogeneity among the included studies (I^2^ = 99.67 %).

### 3.1 Description of studies

Details of the individual studies such as the risk of exposure, study site, sample size, mean age of the participants, and mean BLLs reported are described in Table 1. The included studies reported 23 entries with known (high-risk) exposure. Nine of these entries involving community participants reported average BLL ranging between 7.92 and 28.00 µg/dL. These studies identified potential Pb exposure from cosmetics (viz. surma and sindoor), Pb-based paints, residence/schooling adjacent to Pb-based industries/mines, and occupational Pb exposure among the parents. The remaining fourteen entries involved participants visiting the hospital for Pb exposure-related symptoms viz. neurodevelopmental and neurobehavioral symptoms, seizure, autism spectrum disorder, abdominal pain, etc. The average BLL among the latter group ranged between 5.32 and 42.6 µg/dL. Lastly, 42 entries reporting BLL among children with unknown (low-risk) exposure observed average BLL ranging between 0.4 and 18.6 µg/dL. About 30, 21 & 14 entries are available for later than 2010, between 2000 – 2010 and before 2000.

### 3.2 Risk of Bias assessment

Detail risk of bias potentially involved in the participant definition, selection, exposure, and outcome assessment is described in Table 2. To summarize, 76.47 %, 74.51 %, 82.35 %, 82.35 %, and 80.39 % of included studies carried high risk of bias in participant definition, representativeness of participants during recruitment, during the selection of participants, exposure assessment, and reporting the non-response rate, respectively. Individual studies received 0–4 out of total of 8 stars with an average of 1.04 star.

**Table 2:** Risk of bias.

Author & year	Citation	Is the case definition adequate	Representativeness of the cases	Selection of Participants	Exposure Assessment	Non- Response rate	Total stars
Lu et al., 2024	[38]	*	*	-	-	-	2
Sahu et al., 2024	[39]	*	*	-	-	-	2
Kumar et al., 2023	[40]	*	*	-	-	-	2
Abinaya et al., 2023	[41]	*	*	-	-	-	2
Mahdi et al., 2023	[42]	*	*	-	-	-	2
Brown et al., 2022	[43]	-	-	*	*	-	2
Ramesh et al., 2022	[44]	*	-	*	*	*	4
Rawat et al., 2022	[45]	-	-	*	-	-	1
Shekhawat et al., 2021	[46]	*	-	-	-	*	2
Malavika L et al., 2021	[47]	-	-	-	-	-	0
Ghosh et al., 2021	[28]	*	-	-	-	*	2
Ansari et al., 2020	[48]	-	-	-	-	-	0
Koshy et al., 2020	[49]	*	-	-	-	*	2
Rashid et al., 2019	[35]	-	-	-	-	-	0
Goel and Chowgule, 2019	[50]	-	-	-	-	-	0
Sehgal, 2019	[30]	-	-	-	-	*	1
Santra et al., 2019	[51]	-	-	-	-	-	0
Maheswari et al., 2018	[52]	-	-	-	-	-	0
Chaudhary et al., 2018	[53]	-	*	-	-	*	2
Pratinidhi et al., 2014	[54]	-	-	-	-	-	0
Kalra et al., 2013	[55]	-	-	-	-	-	0
Goswami, 2013	[14]	-	-	-	*	-	1
Patel et al., 2011	[56]	-	-	-	-	-	0
Palaniappan et al., 2011	[57]	-	-	*	-	*	2
Srinivasa Reddy et al., 2011	[58]	-	-	-	-	-	0
Hegde et al., 2010	[59]	-	-	-	-	-	0
Choudhari et al., 2010	[32]	-	-	*	*	-	2
Chaudhary and Sharma, 2010	[33]	-	*	-	*	-	2
Ahamed et al., 2010	[15]	-	-	-	-	-	0
Patel and Athawale, 2009	[60]	-	-	-	-	-	0
Patel and Prabhu, 2009	[60]	-	-	-	-	-	0
Ahamed et al., 2008	[61]	-	-	*	-	-	1
Ahamed et al., 2007	[62]	-	-	*	-	-	1
Zimmermann et al., 2006	[31]	-	-	*	-	*	2
Jain and Hu, 2006	[34]	-	-	-	-	*	1
Nichani et al., 2006	[63]	*	-	-	*	-	2
Mahajan et al., 2005	[64]	-	-	-	-	-	0
Belliniger et al., 2005	[11]	-	*	-	-	-	1
Kalra et al., 2003	[65]	-	*	-	-	-	1
D'Souza et al., 2002	[66]	-	*	-	-	-	1
Mukherjee et al., 2002	[67]	-	-	-	*	-	1
Patel et al., 2001	[68]	-	*	-	*	*	3
Tripathi et al., 2001	[36]	-	-	-	-	-	0
Srivastava et al., 2001	[69]	*	-	-	-	-	1
Raghunath et al., 2000	[70]	*	-	-	-	-	1
Kaul, 1999	[71]	-	-	-	-	-	0
Kumar et al., 1998	[72]	-	-	-	-	-	0
Raghunath et al., 1997	[73]	-	*	-	*	-	2
Gogte et al., 1991	[74]	-	-	-	-	-	0
Khandekar et al., 1987	[75]	-	*	-	-	-	1
Kaul and Kaul, 1986	[76]	-	-	*	-	-	1
Percentage of risk of bias		76.47	74.51	82.35	82.35	80.39	

Notably, due to factors such as the lack of reporting on random participant selection and the recruitment of participants from hospital settings, these studies exhibited a high risk of bias regarding the representativeness of community samples. Consequently, the reported BLLs from these studies may not accurately reflect community-level exposure. Furthermore, the vast majority of studies (n = 35) did not report conducting quality checks for their analytical methods, which could potentially compromise the reliability of the study outcomes (Table 2).

### 3.3 Pooled BLLs of Indian Children

The pooled BLL among Indian children is estimated to be 10.4 (95 % CI: 9.55–11.2) µg/dL with unacceptably high heterogeneity among the included studies (*I*^2^ = 99.67 %) (Fig. 1, Table 3). Galbraith plot revealed heterogeneity among the studies included, in addition to identifying the outliers (Supplementary Fig. 2). Individual observations are consistent with the whole group analysis, as evident by leave-one-out analysis, i.e. the pooled observation did not significantly change by the exclusion of any particular study (Supplement Figure 3). Bubble plot of BLL across time revealed a trend of declining BLLs over the time, and further studies with relatively larger sample sizes are reported during the recent times as compared with before the 1990s (Supplement Figure 4). Although subgroup analysis by the period of data collection i.e. before 2000 *vs.* between 2000 and 2010 *vs* after 2010 revealed significant differences (*p* = 0.01) over the 3-time points, cumulative meta-analysis did not reveal a consistent direction of change in BLLs among the Indian children (Supplement Figs. 5 and 6). The funnel plot suggested the majority of studies carried relatively lower standard errors (∼ < 3 units), albeit with the potential presence of publication bias as studies reporting the presence of BLLs are likely to be published (Supplement Figure 7).

**Table 3:** Summary of the results.

Group & sub- groups	Year	N	BLLs in µg/dL			*I*^2^ (%)
			Pooled mean level	Lower limit of the 95 % Confidence Interval	Upper limit of the 95 % Confidence Interval	
All studies	Before 2000	14	12.5	10.8	14.2	98.76
	2000–10	21	10.6	9.37	11.9	98.03
	After 2010	30	9.28	7.92	10.6	99.83
	Total	65	10.4	9.55	11.2	99.67
High risk	Before 2000	4	16.8	11.8	21.9	94.56
	2000–10	8	11.9	9.2	14.5	95.05
	After 2010	11	15.2	12.1	18.4	98.28
	Total	23	14.3	12.3	16.2	97.54
Low risk	Before 2000	10	10.9	9.09	12.8	98.94
	2000–10	13	10.1	8.54	11.7	98.62
	After 2010	19	6.64	4.97	8.32	99.89
	Total	42	8.71	7.71	9.71	99.78

*I*^*2*^ = heterogeneity

### 3.4 Subgroup analysis of BLLs of Indian Children

**Fig. 2:**
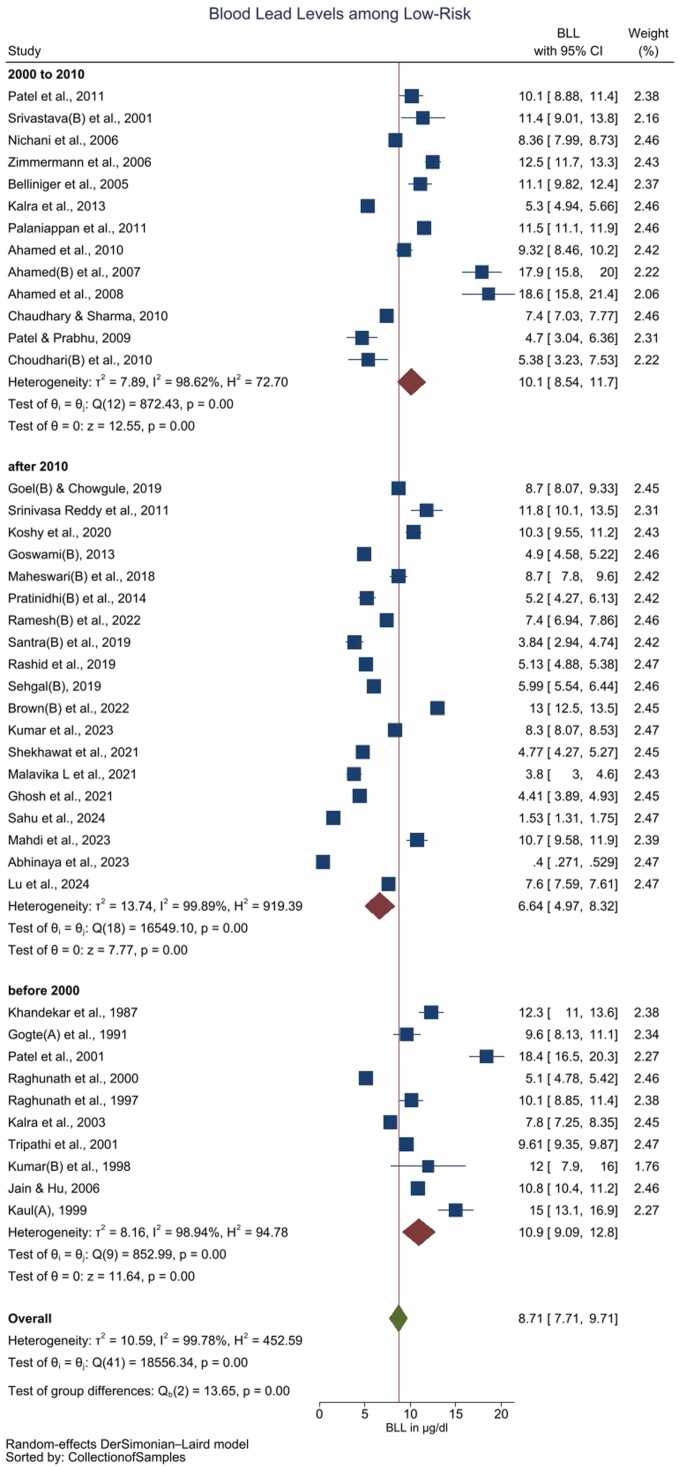
Forest plot of the subgroup analysis (decade) of studies reporting BLL among children with low risk / unknown Pb exposure (Legends / footnotes) Sub group analysis evaluating the influence of study publication (i.e. before 2000 vs. between 2000 and 2010 vs. after 2010) on the results.

The pooled BLL among Indian children with a high-risk of Pb exposure is estimated as 14.3 (95 % CI: 12.3–16.2) µg/dL with high heterogeneity (*I*^2^ = 97.54 %) (Supplement Figure 8, Table 3). In contrast pooled BLL among the children with no known (low risk of) exposure to Pb is 8.71 (95 % CI: 7.71–9.71) µg/dL with high heterogeneity among the included studies (*I*^2^ = 99.78 %) (Fig. 2, Table 3). Bubble plot of BLL across time revealed a steeper decline of BLLs over the period of time among the low-risk group as compared with the high-risk group (Supplement Figure 9 and 10). Sub-group analysis by the period of the data collection i.e. prior to 2000 *vs.* between 2000 and 2010 *vs.* after 2010, revealed significant differences (*p* < 0.01) over the 3-time points (Figure supplement Figure 7). The low-risk group, but not the high risk group exhibited a pattern of reduction in BLLs during these 3-time points (Fig. 2, Supplement Figure 11 and Table 3). The cumulative meta-analysis failed to reveal a consistent direction of changes in the BLLs (Supplement Figure 12 and 13).

Most of the included studies recruited participants from urban locations, with the exception of Hegde et al. [59], Choudhari et al. [32], Chaudhary and Sharma [33], and Lu et al. [38]. The pooled blood lead levels (BLL) for urban participants were significantly higher compared to those reported in studies with non-urban participants (Supplementary Figure 14).

The included studies utilized venous, capillary, and umbilical cord blood samples for BLL measurement. Subgroup analysis did not reveal statistically significant differences between these sample types; however, pooled BLL levels from umbilical cord blood were relatively lower compared to those from capillary or venous blood samples (Supplementary Figure 15).

A wide range of analytical methods were employed to estimate BLL, including anodic stripping voltammetry (ASV), differential pulse ASV, point-of-care ASV, atomic absorption spectrophotometry (AAS), flameless AAS, graphite furnace AAS, colorimetry, inductively coupled plasma atomic emission spectroscopy (ICP-AES), inductively coupled plasma mass spectrometry (ICP-MS), and inductively coupled plasma optical emission spectroscopy (ICP-OES). Subgroup analysis did not indicate significant differences in BLL measurements across these methodologies (Supplementary Figure 16).

The majority of the included studies involved children across a wide age spectrum, while a few specifically focused on newborns and others on children under 5 years of age. Subgroup analysis of these three age groups did not reveal statistically significant differences in BLL. However, newborns showed relatively lower pooled BLL compared to the other age groups (Supplementary Figure 17).

## 4 Discussion

The current study aimed at estimating the pooled BLLs among Indian children aged ≤ 14 years, by systematic review and meta-analysis of published literature. The present study observed pooled mean BLLs of 10.4 (95 % CI: 9.55–11.2) µg/dL with a trend of gradual reduction during the last three decades. Overall pooled mean BLL of participants with a high-risk of Pb exposure is higher than that of the children with no known (low-risk of) exposure to Pb. Children with no known exposure to Pb exhibited a time trend of a gradual reduction in BLLs, while no such time trend is observed among the children with a high risk of Pb exposure. The current review observed high heterogeneity among the included studies.

A complete ban on usage of leaded petrol since 2000 was a historic event in India, towards curbing airborne Pb exposure. The current review demonstrated a gradual decline in BLL post-ban of the leaded petrol, suggesting that it is an effective intervention towards reducing Pb burden. The observations are consistent among children with no known (low risk of) Pb exposure, strengthening the effectiveness of the intervention. Interestingly the number of studies reporting BLL > 10 µg/dL in children with no known/low risk of Pb exposure also decreased gradually, with twelve of the thirteen studies published after 2010 observed a mean BLL < 10 µg/dL. However, the Pb exposure among the high risk group is beyond leaded petrol, therefore no such trend was observed in the latter group. Pb exposure among the high risk group were primarily due to cosmetics (viz. surma and sindoor), Pb-based paints, residence/schooling adjacent to Pb-based industries/mines, adulterated spices, complimentary alternative medicinal preparations containing Pb, and occupational Pb exposure among the parents. Hence, the high risk group require specific interventions, beyond ban in leaded petrol to prevent / reduce the Pb exposure.

Ericson et al., reported pooled BLL of 6.86 μg/dL (95 % CI: 4.38–9.35) in Indian children by systematically pooling seventeen reports available from 2010 to 2018 from a single repository [21]. In addition, the cited study attempted to contrast the BLL of children residing in urban (16 reports) and rural (single report) locations. However, current study reviewed the entire literature across the three repositories from their respective inception to August-01, 2024. Further, current review categorized the participants based on their potential Pb exposure as known / high risk group and the unknown/low risk of Pb exposure, to facilitate the prioritizing of the intervention group. Additionally, current observations highlighted the effect of national policy (i.e. ban of leaded petrol in India) towards reducing environmental Pb burden by time-trend analysis, and observed that the overall decline in the BLL.

The present study systematically reviewed the Indian studies reporting BLL among children aged ≤ 14 years. Our observations are in agreement with the earlier systematic review reports despite pooling observations from additional primary studies [21]. The findings of this systematic review should be interpreted with caution, considering several inherent limitations associated with systematically pooling primary observations. One of the key challenges is the definition of low- and high-risk exposure groups, as it relies on reported sources of lead (Pb) exposure within individual studies. While we classified studies as "low-risk" when no known Pb exposure was reported, some studies in this category still observed blood lead levels (BLL) exceeding 10 µg/dL, indicating potential unrecognized or unreported exposure sources.

Additionally, pooling BLL data from different blood sample types, including capillary and venous blood, introduces variability. Although venous blood is considered the gold standard for lead measurement, several studies reported BLL using capillary samples, which may be prone to external contamination. While our subgroup analysis did not reveal statistically significant differences between sample types, this variability remains a methodological concern.

Another critical limitation is the inclusion of studies with varying levels of bias, particularly observational and cross-sectional designs, which are inherently susceptible to confounding and selection bias. Many studies lacked detailed reporting on sample collection protocols, laboratory quality controls, or adjustments for potential confounders, which could impact the reliability of the pooled estimates. Furthermore, a large proportion of the included studies did not conduct quality checks on their analytical methods, potentially influencing outcome accuracy.

The lack of precise information on sample collection dates is another major limitation. Since BLLs are influenced by environmental regulations and public health interventions over time, tracking temporal trends is crucial. However, when sample collection dates were unavailable, we used the publication year as a proxy, which may not accurately reflect the actual timing of data collection. This introduces uncertainties in assessing the impact of national policies, such as the ban on leaded petrol and other Pb mitigation efforts.

Finally, the heterogeneity among included studies—in terms of study design, participant selection, geographic representation, and analytical methods—further complicates interpretation. While subgroup and meta-regression analyses were conducted to explore sources of heterogeneity, the limited number of studies available for these analyses restricted our ability to draw definitive conclusions.

Despite these limitations, this review provides a critical synthesis of available data on BLL trends among Indian children, offering valuable insights into the effectiveness of national programs aimed at reducing Pb exposure. Future research should focus on improving study design rigor, ensuring standardized BLL measurement protocols, and strengthening biomonitoring efforts to generate high-quality, representative data for more precise risk assessment.

## 5 Conclusions

Pooled evidence suggests that the mean BLL of Indian children is higher than the reference level. The findings of this systematic review indicate a gradual decline in blood lead levels (BLLs) over time, coinciding with national efforts such as the ban on leaded petrol. However, given the inherent limitations of the studies reviewed—such as variability in study design, data quality, and potential biases—these results should be interpreted with caution. Rather than serving as definitive evidence of the effectiveness of national programs, this review primarily establishes a body of evidence on BLL trends and highlights existing gaps in data quality and reporting. Our analysis further underscores that BLLs remain higher among children with known/high-risk Pb exposure compared to those with unknown/low-risk exposure, reinforcing the need for targeted interventions. In light of the limited availability of high-quality studies assessing Pb exposure, we strongly recommend the establishment of a nationwide human biomonitoring program with periodic assessments every 2–3 years. Such a program would provide systematic data on BLL trends, identify high-risk populations, and inform evidence-based public health interventions. Implementing systematic biomonitoring efforts, complemented by targeted longitudinal research, would provide a robust evidence base for policy-making and interventions to reduce lead exposure During such studies, oversampling of low-income and high-risk groups is crucial to address disparities in lead exposure across India. While longitudinal studies by academic institutions provide valuable insights, their limited scope underscores the need for broader, population-wide surveillance frameworks. Moving forward, national efforts should focus not only on reducing Pb exposure but also on strengthening surveillance mechanisms, ensuring standardized exposure assessment methodologies, and addressing research gaps. A more systematic approach to data collection and policy implementation will allow for a more robust evaluation of intervention effectiveness while guiding future public health strategies to mitigate lead exposure in children, particularly in high-risk populations.

## Ethics declaration

The study findings are based on review of literature and no primary data from either humans or animals are included in the current study, hence the ethical approval for the present study is waivered.

## Ethics approval

The study is a systematic review and adhered to the relevant ethical aspects.

## Research involving human participants

The study is a systematic review and adhered to the relevant ethical aspects. The study was approved from the Institute’s ethics committee before initiating and all authors have ensured to adhere to the ethical guidelines during execution of the study.

## Author’s contributions

All authors have sufficiently contributed to the study conception and execution. Details of specific contribution is listed below.

## Consent for publication

The authors have obtained necessary permissions and consent from the participants, respective institutions towards publishing this document

## Funding

The study was executed with institutional funds and the authors declare than no external funds, grants or other support were received during the preparation of this manuscript.

## CRediT authorship contribution statement

**Raju Nagaraju:** Writing – review & editing, Investigation, Data curation. **Yadav Geetika:** Writing – review & editing, Conceptualization. **Ravibabu Kalahasthi:** Writing – review & editing, Methodology, Conceptualization. **Dhananjayan Venugopal:** Writing – review & editing, Methodology, Conceptualization. **Balachandar Rakesh:** Writing – original draft, Project administration, Investigation, Formal analysis, Data curation, Conceptualization. **Bagepally Bhavani Shankara:** Writing – review & editing, Methodology, Formal analysis, Conceptualization. **Upadhyay Kuldip:** Writing – original draft, Project administration, Methodology, Investigation, Formal analysis, Data curation, Conceptualization. **Das Santasabuj:** Writing – review & editing, Methodology, Conceptualization. **Ravichandran Beerappa:** Writing – review & editing, Methodology, Investigation, Conceptualization.

## Declaration of Competing Interest

The authors declare that they have no known competing financial interests or personal relationships that could have appeared to influence the work reported in this paper. Rakesh B reports was provided by ICMR - National Institute of Occupational Health. Rakesh B reports a relationship with ICMR - National Institute of Occupational Health that includes: employment. Rakesh B has patent pending to None. None to declare If there are other authors, they declare that they have no known competing financial interests or personal relationships that could have appeared to influence the work reported in this paper

## Data Availability

Data will be made available on request.
