# Supplementary material for: Estimation of the pooled mean blood lead levels of Indian children: Evidence from systematic review and meta-analysis
Source: Toxicol Rep. 2025 Feb 25;14:101975. doi: 10.1016/j.toxrep.2025.101975 (PMC11914758; doi:10.1016/j.toxrep.2025.101975)
Supplement: Supplementary file 1 — Supplementary material [file mmc1.docx]

Contents

[Supplement Figure 1: PRISMA 2020 flow chart 2](#_Toc188632675)

[Supplementary Figure 2: Galbraith plot for BLL 3](#_Toc188632676)

[Supplementary Figure 3: leave-one-out plot of the studies included in the review 4](#_Toc188632677)

[Supplementary Figure 4: Bubble plot of the studies included in the review 5](#_Toc188632678)

[Supplementary Figure 5: Cumulative plot of all studies included in the review 6](#_Toc188632679)

[Supplementary Figure 6: Forest plot of the subgroup analysis (decade) of studies reporting BLL among children 7](#_Toc188632680)

[Supplement Figure 7: Funnel plot of studies included in the current systematic review 8](#_Toc188632681)

[Supplement Figure 8: Forest plot of the subgroup analysis (decade) of studies reporting BLL among children with high risk / known Pb exposure 9](#_Toc188632682)

[Supplementary Figure 9: Bubble plot of the studies reporting BLL among children with low risk / unknown Pb exposure 10](#_Toc188632683)

[Supplementary Figure 10: Bubble plot of the studies reporting BLL among children with high risk / known Pb exposure 11](#_Toc188632684)

[Supplementary Figure 11: Mean BLL of studies (all, high risk and low risk) in the order of the year of publication. 12](#_Toc188632685)

[Supplementary Figure 12: Cumulative plot of all studies reporting BLL among children with high risk / known Pb exposure 13](#_Toc188632686)

[Supplementary Figure 13: Cumulative plot of all studies reporting BLL among children with low risk / unknown Pb exposure 14](#_Toc188632687)

[Supplementary Figure 14: Forest plot of the subgroup analysis (residence location) of studies reporting BLL among all children 15](#_Toc188632688)

[Supplementary Figure 15: Forest plot of the subgroup analysis (type of sample) of studies reporting BLL among all children 16](#_Toc188632689)

[Supplementary Figure 16: Forest plot of the subgroup analysis (analytical method of Pb estimation) of studies reporting BLL among all children 17](#_Toc188632690)

[Supplementary Figure 17: Forest plot of the subgroup analysis (analytical method of Pb estimation) of studies reporting BLL among all children 18](#_Toc188632691)

[Supplementary table 1a: Search strategy at pubmed medline digital library 19](#_Toc188632692)

[Supplementary table 1b: Search strategy at embase digital library 19](#_Toc188632693)

[Supplementary table 1c: Search strategy at scopus digital library 19](#_Toc188632694)

[SOP for risk of bias assessment adopted from OHRI-Newcastle-Ottawa quality assessment scale 20](#_Toc188632695)

[**PRISMA 2020 abstract checklist items** 21](#_Toc188632696)

[**PRISMA 2020 checklist items** 22](#_Toc188632697)

## Supplement Figure 1: PRISMA 2020 flow chart

**Identification of studies via databases and registers**

Records identified from:

Databases (n = 8295)

- PubMed (n = 3959)
- Scopus (n = 3177)
- Embase (n = 1159)
- Lateral search (n = 0)

Registers (n = 0)

Records removed *before screening*:

Duplicate records removed (n = 796)

- Records marked as ineligible by automation tools (n = 536)
- Records removed for other reasons (n = 0)
- Records manually identified as duplicate (n = 260)

**Identification**

Records excluded (n = 7351)

• Involved participants other than children < 14 years (n = 4003)

• Blood lead levels not evaluated (n = 2859)

• Preclinical studies (n = 337)

• Review / letter to editor / perspective / methods (n = 152)

Records screened

(n = 7499)

Reports sought for retrieval

(n = 148)

Reports not retrieved

(n = 0)

**Screening**

Reports excluded (n = 97):

• No values available for extraction (n = 37)

• No full texts (n = 8)

• Reviews / perspectives / letter to editor (n = 24)

• Others (n = 28)

Reports assessed for eligibility

(n = 148)

Studies included in review

(n = 51)

Reports of included studies

(n = 65)

**Included**

*From:*  Page MJ, McKenzie JE, Bossuyt PM, Boutron I, Hoffmann TC, Mulrow CD, et al. The PRISMA 2020 statement: an updated guideline for reporting systematic reviews. BMJ 2021;372:n71. doi: 10.1136/bmj.n71

For more information, visit: <http://www.prisma-statement.org/>

(Legends / footnotes) Flow chart illustrating the number of citations / articles included and excluded at various stages

## Supplementary Figure 2: Galbraith plot for BLL


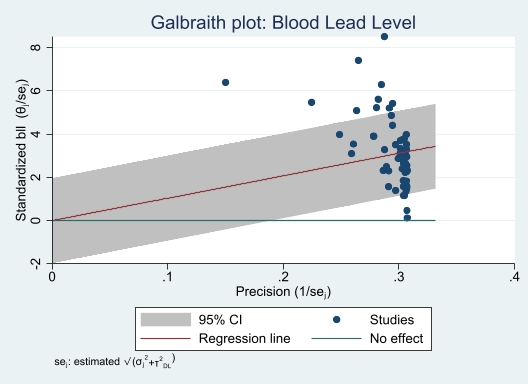


(Legend / footnote) X axis represents the 1/SE of BLL, while y axis represents z-statistics by dividing each estimate by its standard error and y axis represents the precision of the measure (standard error).

## Supplementary Figure 3: leave-one-out plot of the studies included in the review


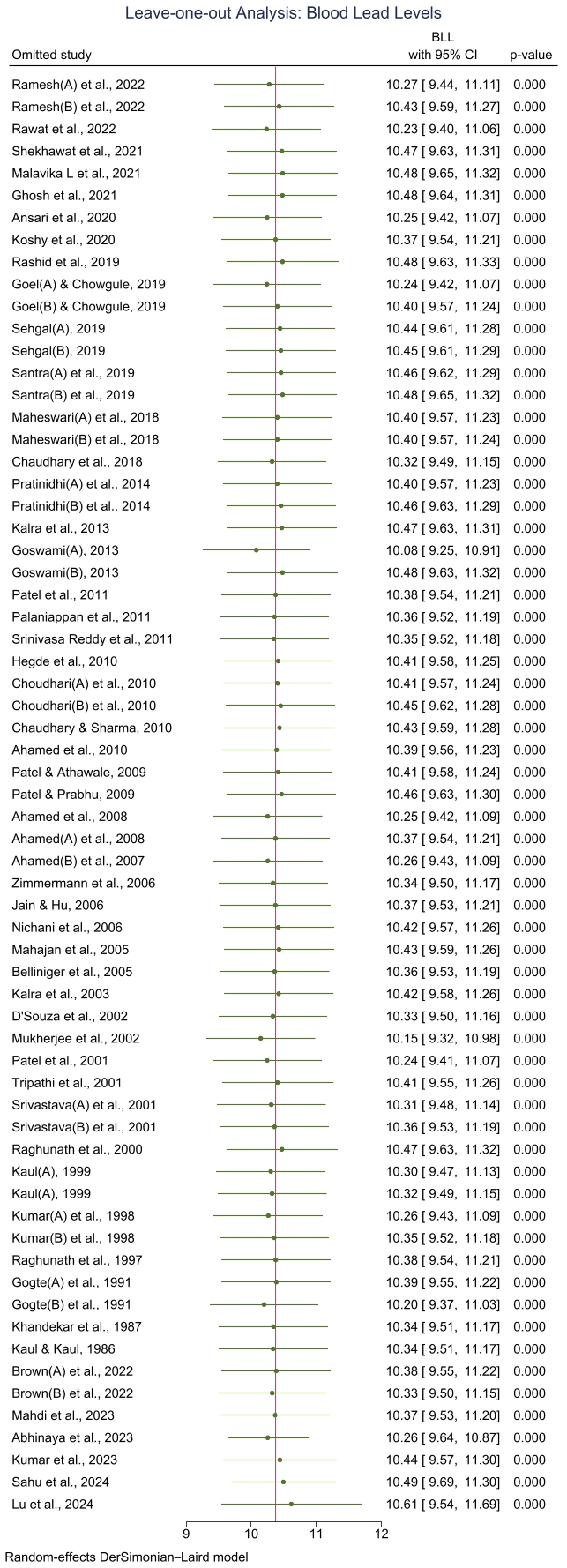


(Legends / footnotes) Mean BLL after excluding each study.

## Supplementary Figure 4: Bubble plot of the studies included in the review


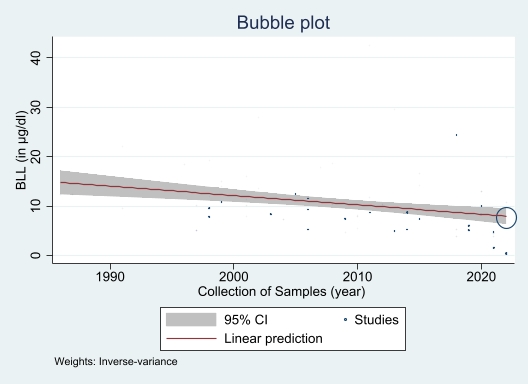


(Legends / footnotes) X axis represents the year of publication, while y axis represents mean BLL reported by the respective study and the size of bubble is relative sample size of the respective study.

## Supplementary Figure 5: Cumulative plot of all studies included in the review


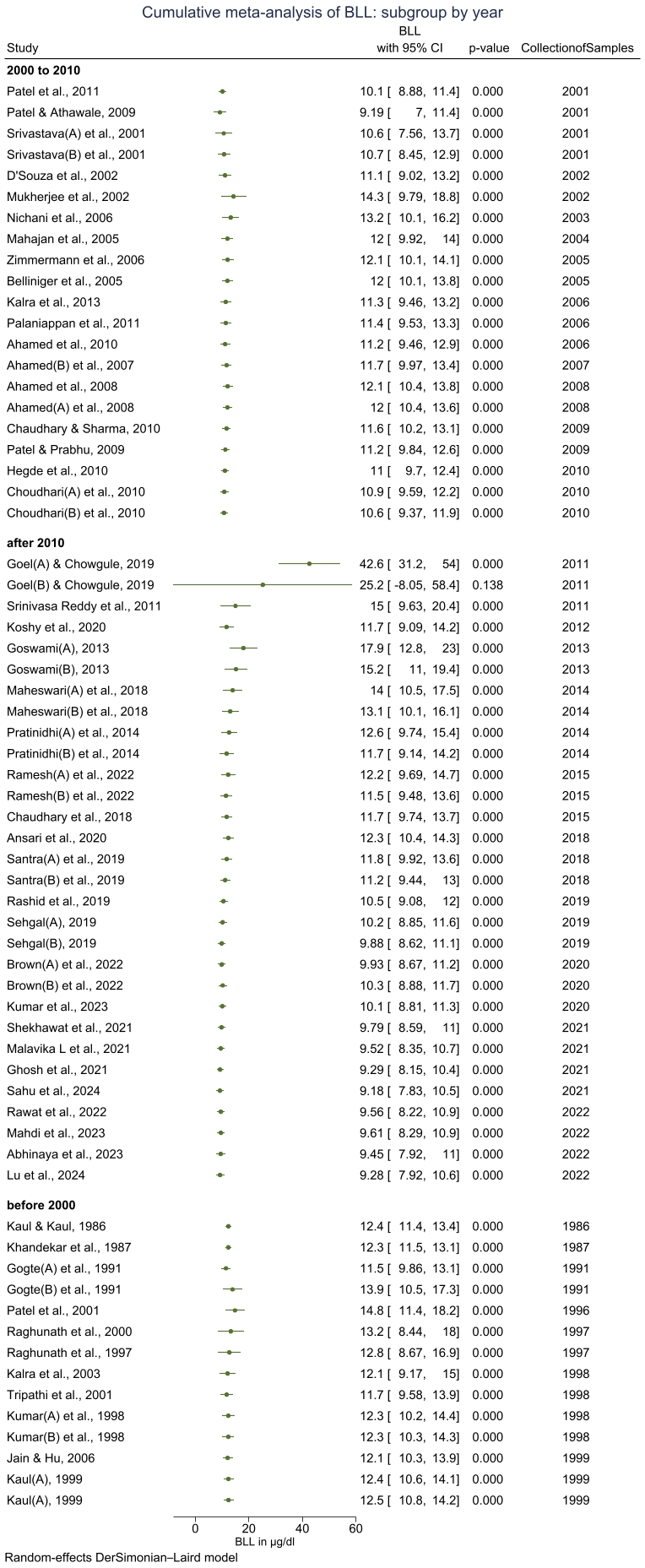


(Legends / footnotes) Mean BLL of studies in the order of the year of publication.

## Supplementary Figure 6: Forest plot of the subgroup analysis (decade) of studies reporting BLL among children


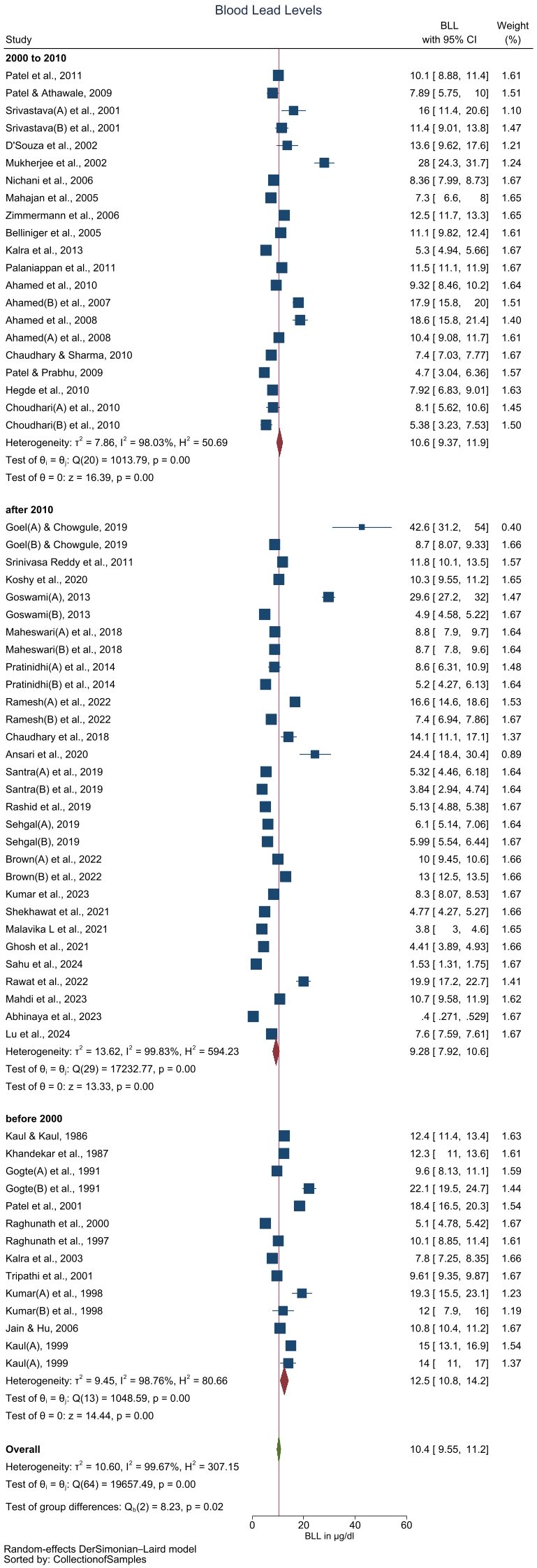


(Legends / footnotes) Sub group analysis evaluating the influence of study publication (i.e. before 2000 vs. between 2000 - 2010 vs. after 2010) on the results

## Supplement Figure 7: Funnel plot of studies included in the current systematic review


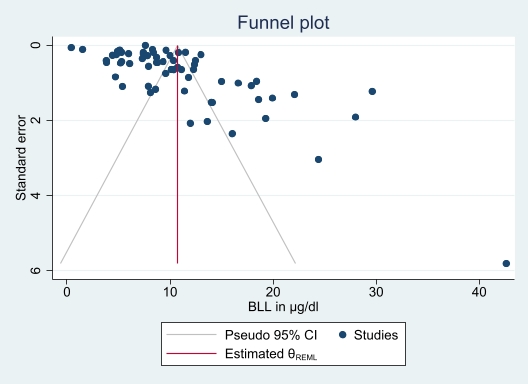


(Legends / footnotes) X axis represents the estimated mean BLL (µg/dL) and the y axis represents the precision of the measure (standard error). Individual dots represent individual studies and their corresponding mean BLL and standard of error.

## Supplement Figure 8: Forest plot of the subgroup analysis (decade) of studies reporting BLL among children with high risk / known Pb exposure


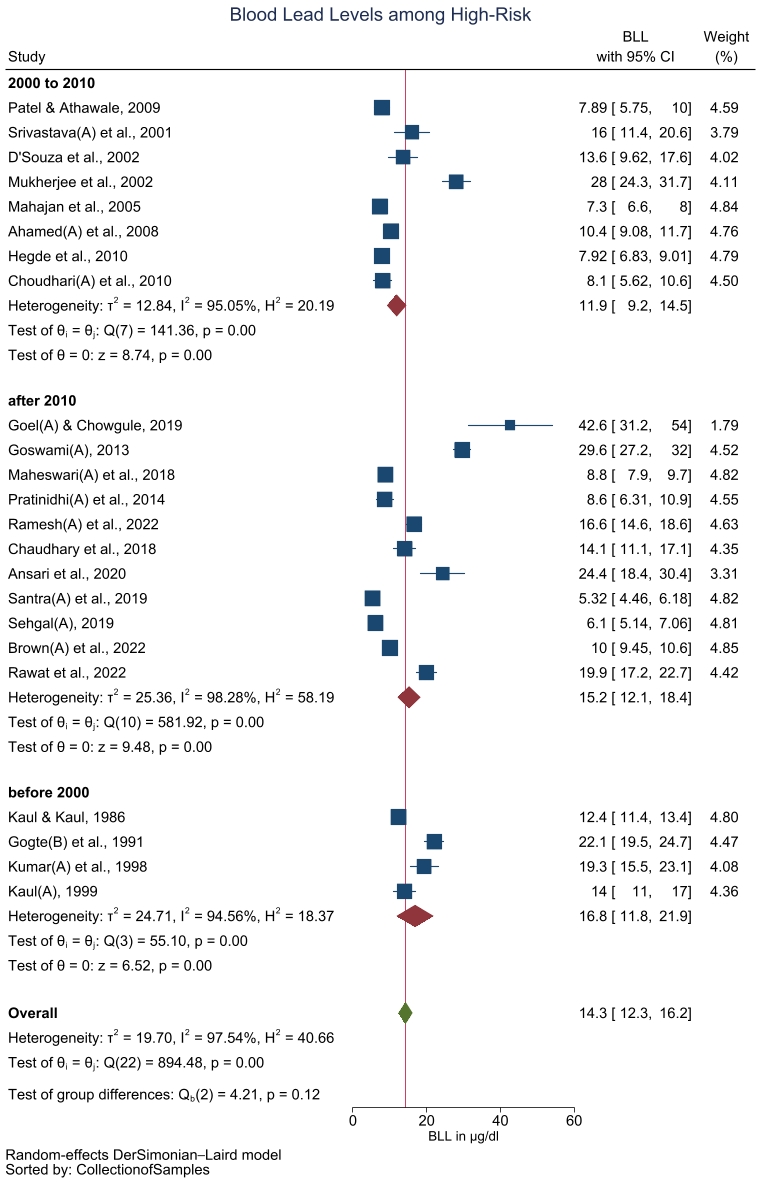


(Legends / footnotes) Sub group analysis evaluating the influence of study publication (i.e. before 2000 vs. between 2000 - 2010 vs. after 2010) on the results

## Supplementary Figure 9: Bubble plot of the studies reporting BLL among children with low risk / unknown Pb exposure


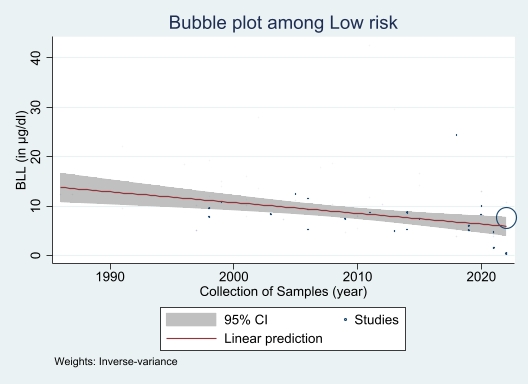


(Legends / footnotes) X axis represents the year of publication, while y axis represents mean BLL reported by the respective study and the size of bubble is relative sample size of the respective study.

## Supplementary Figure 10: Bubble plot of the studies reporting BLL among children with high risk / known Pb exposure


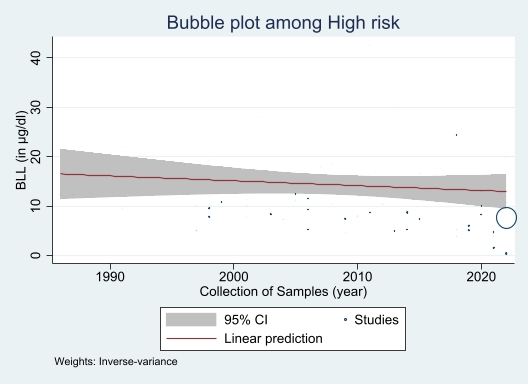


(Legends / footnotes) X axis represents the year of publication, while y axis represents mean BLL reported by the respective study and the size of bubble is relative sample size of the respective study.

## Supplementary Figure 11: Mean BLL of studies (all, high risk and low risk) in the order of the year of publication.


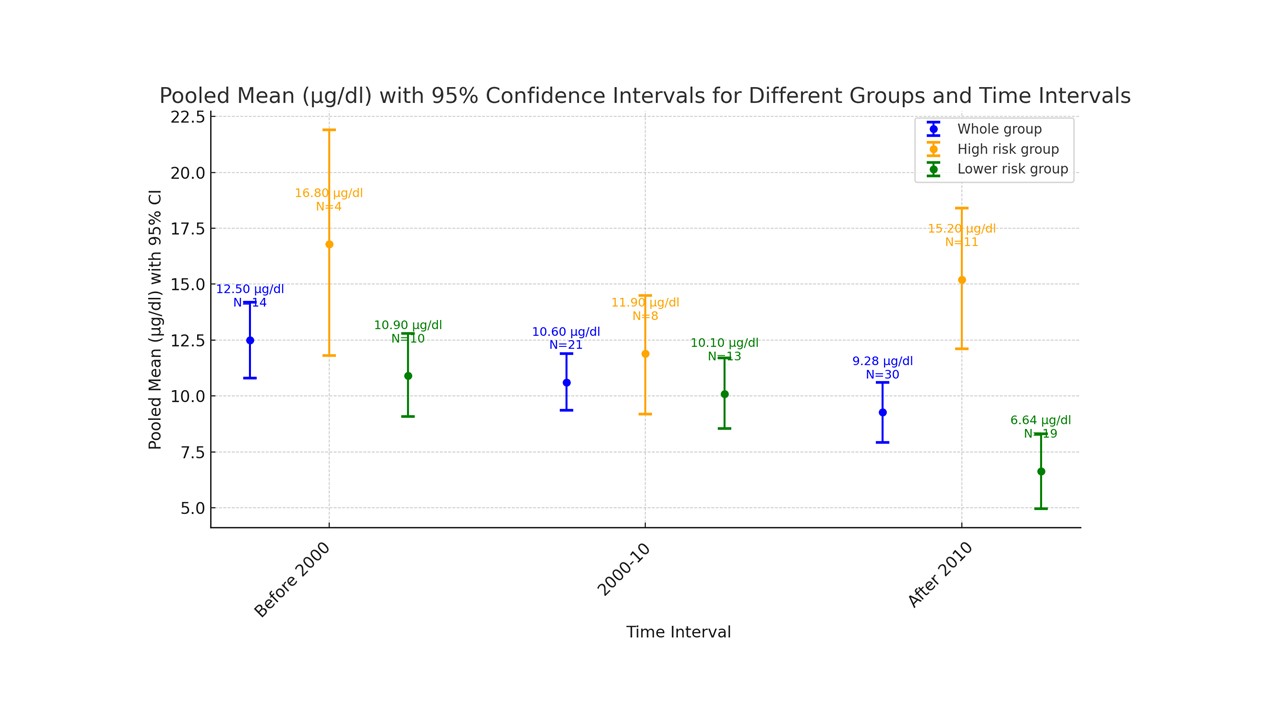


(Legends / footnotes) Whisker (95% CI) and boxplot of the pooled mean blood lead levels of the all studies (blue), studies with high risk (yellow) and low risk (green) of Pb exposure, across the 3 decades.

## Supplementary Figure 12: Cumulative plot of all studies reporting BLL among children with high risk / known Pb exposure


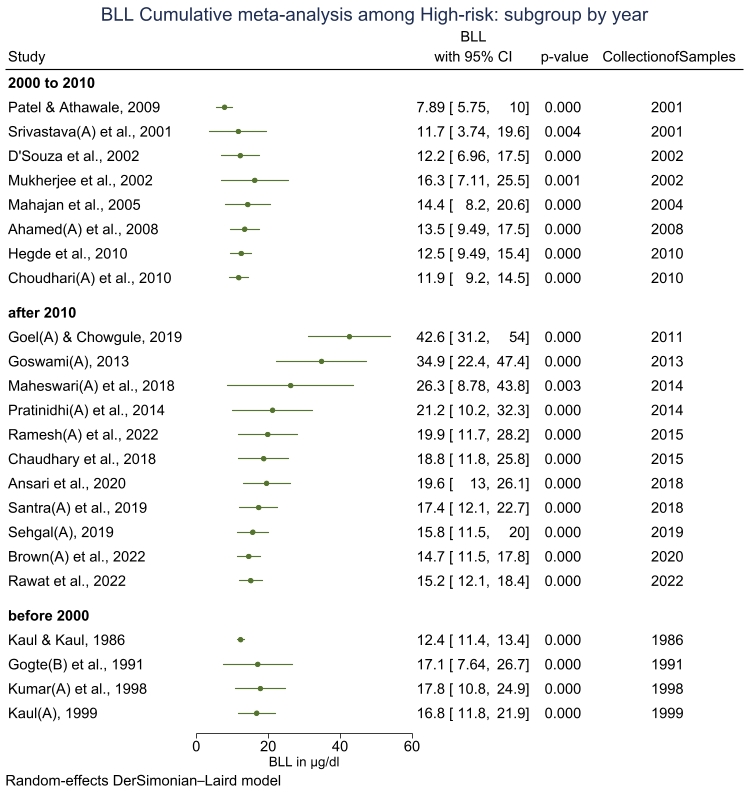


(Legends / footnotes) Mean BLL of studies in the order of the year of publication.

## Supplementary Figure 13: Cumulative plot of all studies reporting BLL among children with low risk / unknown Pb exposure


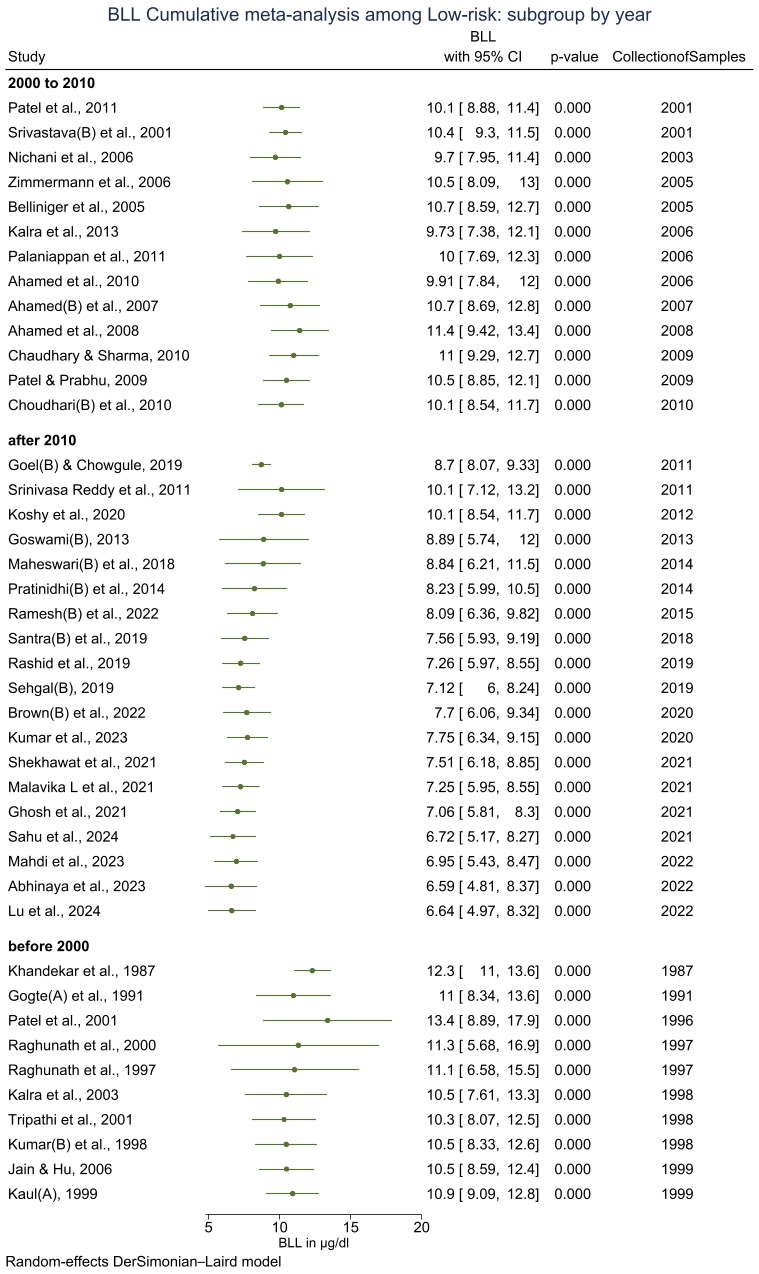


(Legends / footnotes) Mean BLL of studies in the order of the year of publication.

##
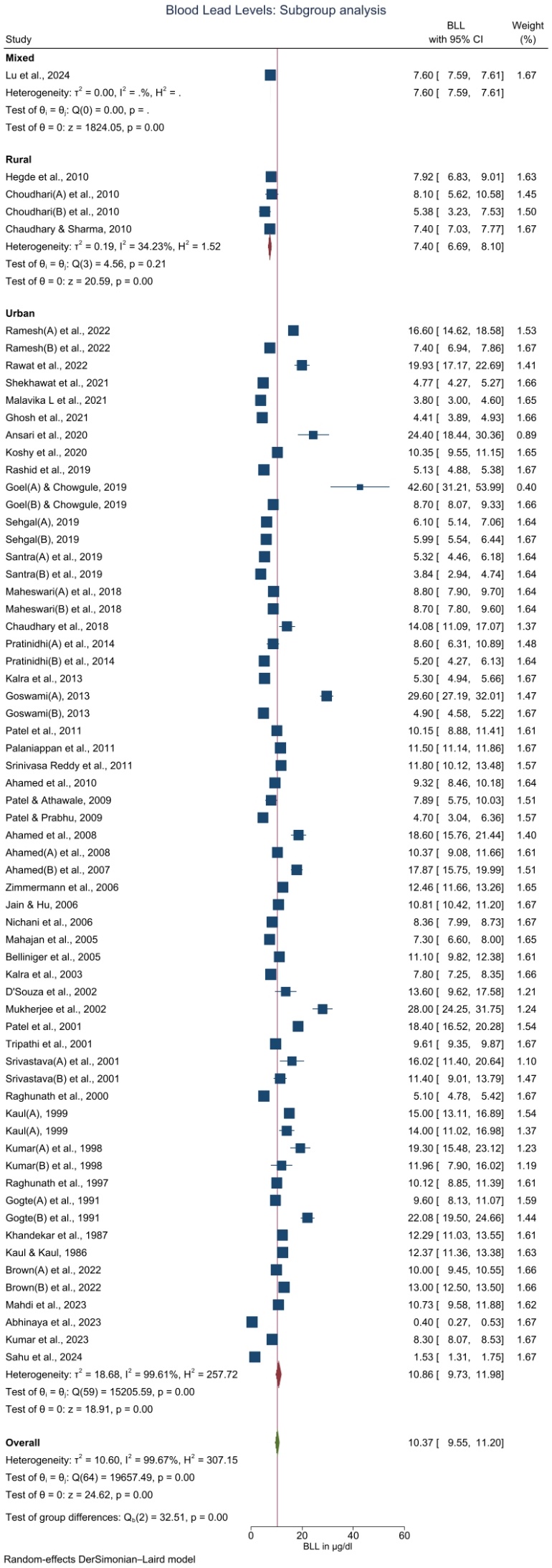
Supplementary Figure 14: Forest plot of the subgroup analysis (residence location) of studies reporting BLL among all children

(Legends / footnotes) Sub group analysis evaluating the influence of place of residence of the participants (i.e. Urban vs. rural vs. mixed) on the results

##
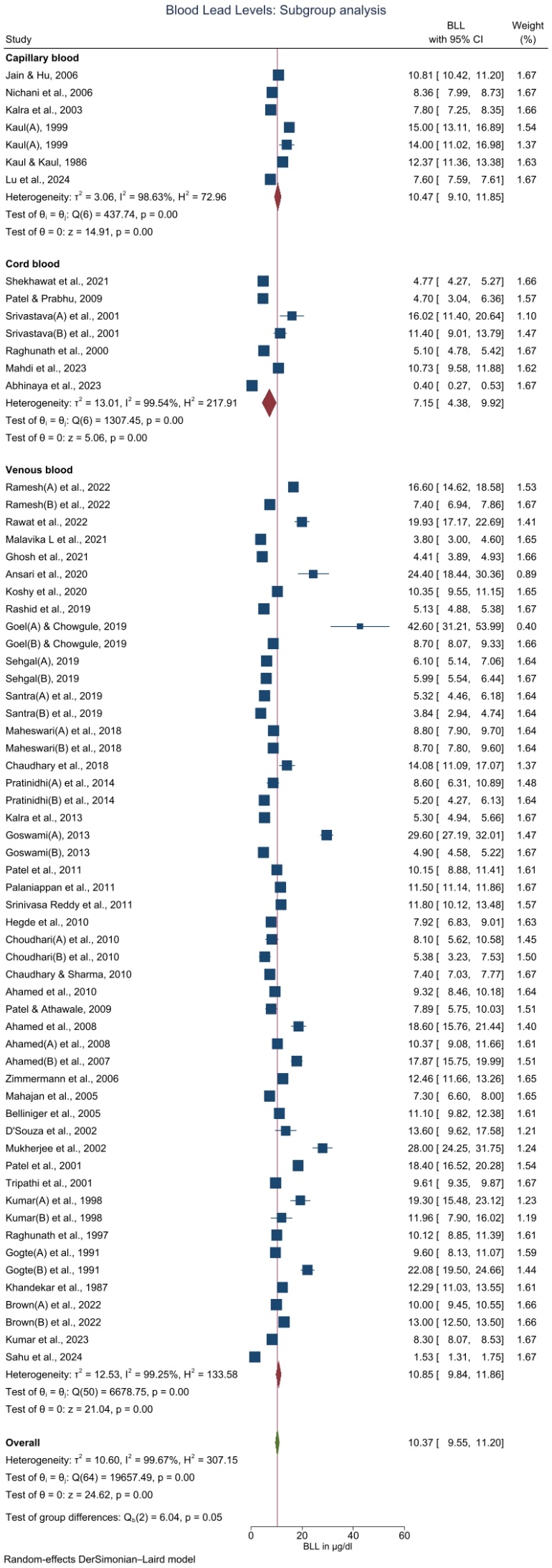
Supplementary Figure 15: Forest plot of the subgroup analysis (type of sample) of studies reporting BLL among all children

(Legends / footnotes) Sub group analysis evaluating the influence of the type of sample collected for BLL estimation (i.e. venous blood vs. capillary blood vs. umbilical cord blood) on the results

##
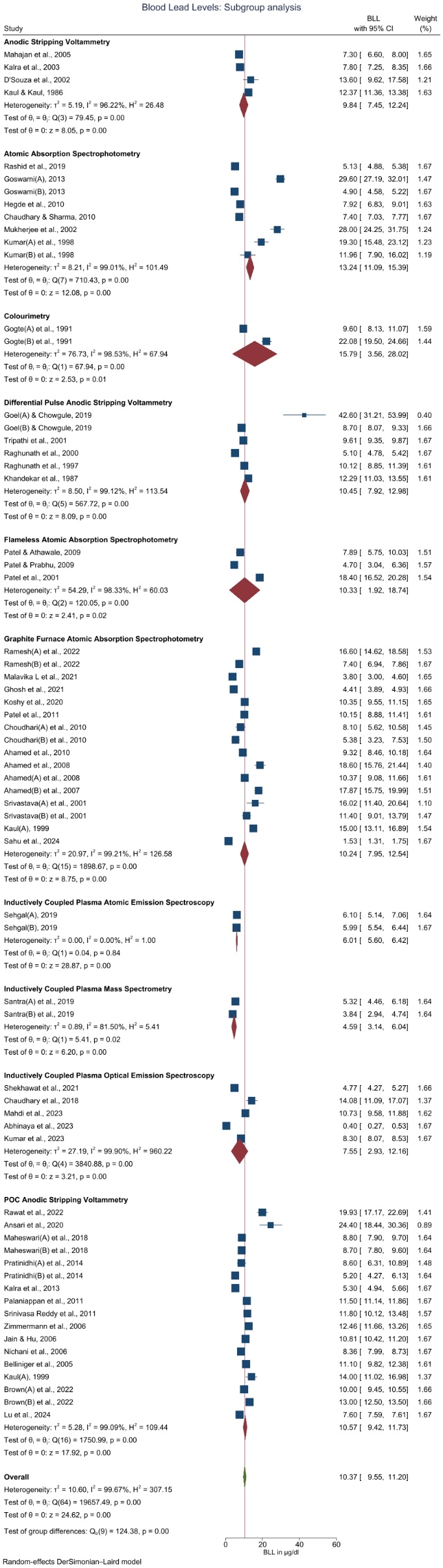
Supplementary Figure 16: Forest plot of the subgroup analysis (analytical method of Pb estimation) of studies reporting BLL among all children

(Legends / footnotes) Sub group analysis evaluating the influence of analytical methods for BLL estimation (i.e. anodic stripping voltammetry (ASV), differential pulse ASV, point-of-care ASV, atomic absorption spectrophotometry (AAS), flameless AAS, graphite furnace AAS, colorimetry, inductively coupled plasma atomic emission spectroscopy (ICP-AES), inductively coupled plasma mass spectrometry (ICP-MS), and inductively coupled plasma optical emission spectroscopy (ICP-OES)) on the results

##
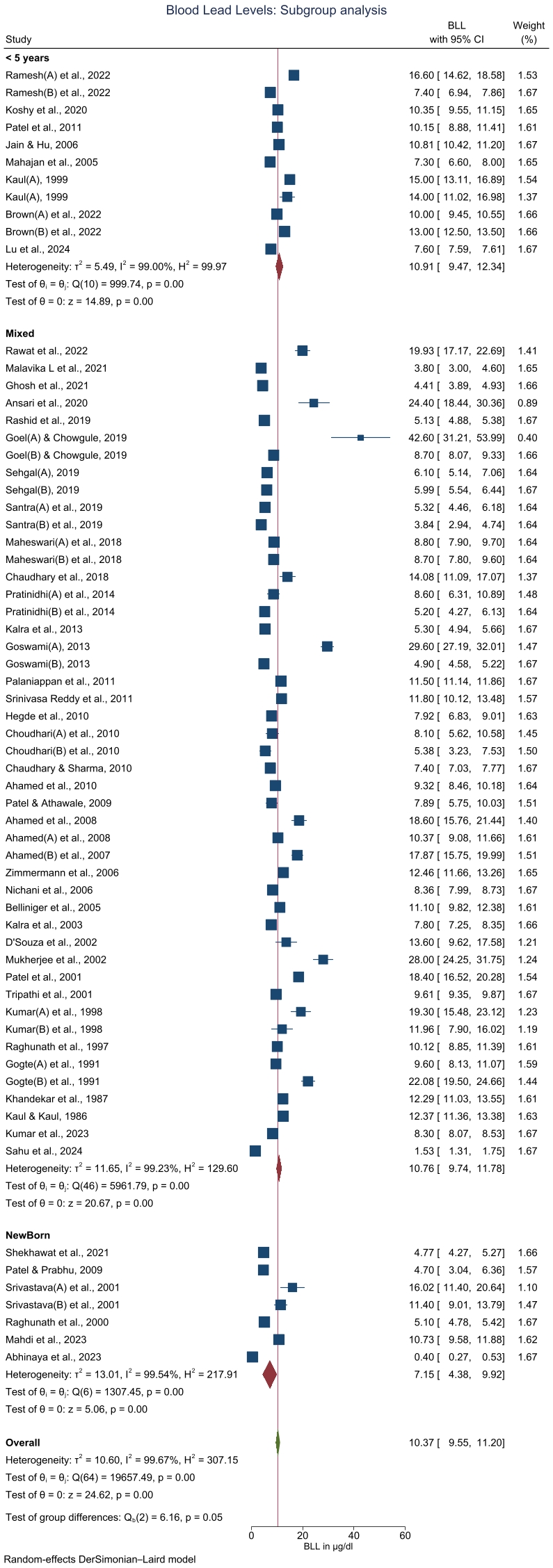
Supplementary Figure 17: Forest plot of the subgroup analysis (analytical method of Pb estimation) of studies reporting BLL among all children

(Legends / footnotes) Sub group analysis evaluating the influence of the age group of children (i.e. < 5 years vs. Mixed vs. New born) on the results

## Supplementary table 1a: Search strategy at pubmed medline digital library

| S.N. | Search terms | Pubmed Keywords | Search hits |
| --- | --- | --- | --- |
| 1 | Blood | blood OR blood[MeSH Terms] | [5,363,365](https://pubmed.ncbi.nlm.nih.gov/?term=blood+OR+blood%5BMeSH+Terms%5D&sort=date&size=100&ac=no) |
| 2 | Lead | lead OR lead[MeSH Terms] | [761,081](https://pubmed.ncbi.nlm.nih.gov/?term=lead+OR+lead%5BMeSH+Terms%5D&sort=date&size=100&ac=no) |
| 3 | India | India | [731,288](https://pubmed.ncbi.nlm.nih.gov/?term=India&sort=date&size=100&ac=no) |
| 4 |  | 1 & 2  & 3 | [3,959](https://pubmed.ncbi.nlm.nih.gov/?term=%28%28blood+OR+blood%5BMeSH+Terms%5D%29+AND+%28lead+OR+lead%5BMeSH+Terms%5D%29%29+AND+%28India%29&sort=date&size=100&ac=no) |

## Supplementary table 1b: Search strategy at embase digital library

| S.N. | Search terms | Emabse Keywords | Search hits |
| --- | --- | --- | --- |
| 1 | Blood | blood OR blood [MeSH Terms] | 2,704,128 |
| 2 | Lead | lead OR lead [MeSH Terms] | 1,067,050 |
| 3 | India | India | [1,318,730](https://www.embase.com/) |
| 4 |  | 1 & 2  & 3 | [2,529](https://www.embase.com/) |
| 5 |  | 1 & 2  & 3 AND 'human'/de AND ('article'/it OR 'article in press'/it OR 'letter'/it) | 1159 |

## Supplementary table 1c: Search strategy at scopus digital library

| S.N. | Search terms | Scopus Keywords | Search hits |
| --- | --- | --- | --- |
| 1 | Blood | blood | [8,987,767](https://www-scopus-com.ejournal.mahidol.ac.th/search/history/results.uri?origin=searchhistory&shid=1) |
| 2 | Lead | TITLE-ABS-KEY ( "lead acetate"  OR  "lead tetraoxide"  OR  "lead tetraacetate"  OR  "lead chromate"  OR  "lead phosphate"  OR  "lead oxide"  OR  "lead silicate"  OR  "tetraethyl lead"  OR  "lead poisoning"  OR  "Pb"  OR  "plumbum"  OR  "tetraethyl" ) | [321,141](https://www-scopus-com.ejournal.mahidol.ac.th/search/history/results.uri?origin=searchhistory&shid=2) |
| 3 | India | India | [4,347,542](https://www-scopus-com.ejournal.mahidol.ac.th/search/history/results.uri?origin=searchhistory&shid=4) |
| 4 |  | 1 & 2  & 3 | [3,908](https://www-scopus-com.ejournal.mahidol.ac.th/search/history/results.uri?origin=searchhistory&shid=5) |
| 5 |  | ( blood ) AND ( TITLE-ABS-KEY ( "lead acetate" OR "lead tetraoxide" OR "lead tetraacetate" OR "lead chromate" OR "lead phosphate" OR "lead oxide" OR "lead silicate" OR "tetraethyl lead" OR "lead poisoning" OR "pb" OR "plumbum" OR "tetraethyl" ) ) AND ( india ) AND ( LIMIT-TO ( DOCTYPE , "ar" ) ) AND ( LIMIT-TO ( EXACTKEYWORD , "lead" ) OR LIMIT-TO ( EXACTKEYWORD , "article" ) OR LIMIT-TO ( EXACTKEYWORD , "human" ) ) AND ( LIMIT-TO ( SRCTYPE , "j" ) ) | [3,177](https://www-scopus-com.ejournal.mahidol.ac.th/search/history/results.uri?origin=searchhistory&shid=8) |

## SOP for risk of bias assessment adopted from OHRI-Newcastle-Ottawa quality assessment scale

Note: A study can be awarded a maximum of one star for each numbered item within the Selection and Exposure categories.

Participant selection

1) Is the participant definition adequate?

a) Yes, Children < 14 years with independent validation using birth records or similar records *

 b) Yes, e.g. using verbal confirmation by parents / guardians / teachers or based on self-reports

c) No description

2) Representativeness of the participant cases

a) Randomly chosen participant from the community*

b) Potential for selection biases or not stated

3) Selection of participants

a) Community controls *

b) Hospital controls

c) No description

Exposure assessment

a) Secure record, estimation of lead levels from all potential sources, soil, air, food, water.*

b) Laboratory staff estimating Pb levels blinded to potential sources of Pb exposure *

c) Laboratory staff estimating Pb levels not blinded to potential Pb exposure status

d) Written self-report or medical record only

e) No description

Non-Response rate

a) Reported among the participants *

b) Non respondents not described

| **Section and Topic** | **Item #** | **PRISMA 2020 abstract checklist items** | **Reported (Yes/No)** |
| --- | --- | --- | --- |
| **TITLE** | | |  |
| Title | 1 | Identify the report as a systematic review. | Yes, 1 |
| **BACKGROUND** | | |  |
| Objectives | 2 | Provide an explicit statement of the main objective(s) or question(s) the review addresses. | Yes, 1 |
| **METHODS** | | |  |
| Eligibility criteria | 3 | Specify the inclusion and exclusion criteria for the review. | Yes, 1 |
| Information sources | 4 | Specify the information sources (e.g. databases, registers) used to identify studies and the date when each was last searched. | Yes, 1 |
| Risk of bias | 5 | Specify the methods used to assess risk of bias in the included studies. | Yes, 1 |
| Synthesis of results | 6 | Specify the methods used to present and synthesise results. | Yes, 1 |
| **RESULTS** | | |  |
| Included studies | 7 | Give the total number of included studies and participants and summarise relevant characteristics of studies. | Yes, 2 |
| Synthesis of results | 8 | Present results for main outcomes, preferably indicating the number of included studies and participants for each. If meta-analysis was done, report the summary estimate and confidence/credible interval. If comparing groups, indicate the direction of the effect (i.e. which group is favoured). | Yes, 3 |
| Limitations of evidence | 9 | Provide a brief summary of the limitations of the evidence included in the review (e.g. study risk of bias, inconsistency and imprecision). | Yes, 4 |
| **DISCUSSION** | | |  |
| Interpretation | 10 | Provide a general interpretation of the results and important implications. | Yes, 2 |
| **OTHER** | | |  |
| Funding | 11 | Specify the primary source of funding for the review. | Yes, 2 |
| Registration | 12 | Provide the register name and registration number. | Yes, 2 |

*From:*  Page MJ, McKenzie JE, Bossuyt PM, Boutron I, Hoffmann TC, Mulrow CD, et al. The PRISMA 2020 statement: an updated guideline for reporting systematic reviews. BMJ 2021;372:n71. doi: 10.1136/bmj.n71

For more information, visit: <http://www.prisma-statement.org/>

| **Section and Topic** | **Item #** | **PRISMA 2020 checklist items** | **Location where item is reported** |
| --- | --- | --- | --- |
| **TITLE** | | |  |
| Title | 1 | Identify the report as a systematic review. | Page 1 |
| **ABSTRACT** | | |  |
| Abstract | 2 | See the PRISMA 2020 for Abstracts checklist. | supplement |
| **INTRODUCTION** | | |  |
| Rationale | 3 | Describe the rationale for the review in the context of existing knowledge. | Page 3 |
| Objectives | 4 | Provide an explicit statement of the objective(s) or question(s) the review addresses. | Page 4 |
| **METHODS** | | |  |
| Eligibility criteria | 5 | Specify the inclusion and exclusion criteria for the review and how studies were grouped for the syntheses. | Page 4 |
| Information sources | 6 | Specify all databases, registers, websites, organisations, reference lists and other sources searched or consulted to identify studies. Specify the date when each source was last searched or consulted. | Page 4 |
| Search strategy | 7 | Present the full search strategies for all databases, registers and websites, including any filters and limits used. | Supplement |
| Selection process | 8 | Specify the methods used to decide whether a study met the inclusion criteria of the review, including how many reviewers screened each record and each report retrieved, whether they worked independently, and if applicable, details of automation tools used in the process. | Page 4 & 5 |
| Data collection process | 9 | Specify the methods used to collect data from reports, including how many reviewers collected data from each report, whether they worked independently, any processes for obtaining or confirming data from study investigators, and if applicable, details of automation tools used in the process. | Page 4 & 5 |
| Data items | 10a | List and define all outcomes for which data were sought. Specify whether all results that were compatible with each outcome domain in each study were sought (e.g. for all measures, time points, analyses), and if not, the methods used to decide which results to collect. | Page 5 |
|  | 10b | List and define all other variables for which data were sought (e.g. participant and intervention characteristics, funding sources). Describe any assumptions made about any missing or unclear information. | Page 5 |
| Study risk of bias assessment | 11 | Specify the methods used to assess risk of bias in the included studies, including details of the tool(s) used, how many reviewers assessed each study and whether they worked independently, and if applicable, details of automation tools used in the process. | Page 5 |
| Effect measures | 12 | Specify for each outcome the effect measure(s) (e.g. risk ratio, mean difference) used in the synthesis or presentation of results. | Page 5 |
| Synthesis methods | 13a | Describe the processes used to decide which studies were eligible for each synthesis (e.g. tabulating the study intervention characteristics and comparing against the planned groups for each synthesis (item #5)). | Page 5 |
|  | 13b | Describe any methods required to prepare the data for presentation or synthesis, such as handling of missing summary statistics, or data conversions. | Page 5 |
|  | 13c | Describe any methods used to tabulate or visually display results of individual studies and syntheses. | Page 5 |
|  | 13d | Describe any methods used to synthesize results and provide a rationale for the choice(s). If meta-analysis was performed, describe the model(s), method(s) to identify the presence and extent of statistical heterogeneity, and software package(s) used. | Page 5 |
|  | 13e | Describe any methods used to explore possible causes of heterogeneity among study results (e.g. subgroup analysis, meta-regression). | Page 5 |
|  | 13f | Describe any sensitivity analyses conducted to assess robustness of the synthesized results. | Page 5 |
| Reporting bias assessment | 14 | Describe any methods used to assess risk of bias due to missing results in a synthesis (arising from reporting biases). | Page 6 |
| Certainty assessment | 15 | Describe any methods used to assess certainty (or confidence) in the body of evidence for an outcome. | Page 5 |
| **RESULTS** | | |  |
| Study selection | 16a | Describe the results of the search and selection process, from the number of records identified in the search to the number of studies included in the review, ideally using a flow diagram. | Page 6 |
|  | 16b | Cite studies that might appear to meet the inclusion criteria, but which were excluded, and explain why they were excluded. | Supplement |
| Study characteristics | 17 | Cite each included study and present its characteristics. | Page 6 &7 & table 1 |
| Risk of bias in studies | 18 | Present assessments of risk of bias for each included study. | Page 7 & table 2 |
| Results of individual studies | 19 | For all outcomes, present, for each study: (a) summary statistics for each group (where appropriate) and (b) an effect estimate and its precision (e.g. confidence/credible interval), ideally using structured tables or plots. | Page 7 & 8, Figure 2-4 |
| Results of syntheses | 20a | For each synthesis, briefly summarise the characteristics and risk of bias among contributing studies. | Page 7 & 8 |
|  | 20b | Present results of all statistical syntheses conducted. If meta-analysis was done, present for each the summary estimate and its precision (e.g. confidence/credible interval) and measures of statistical heterogeneity. If comparing groups, describe the direction of the effect. | Page 7 - 8 |
|  | 20c | Present results of all investigations of possible causes of heterogeneity among study results. | Page 7-8 |
|  | 20d | Present results of all sensitivity analyses conducted to assess the robustness of the synthesized results. | Page 7-8 |
| Reporting biases | 21 | Present assessments of risk of bias due to missing results (arising from reporting biases) for each synthesis assessed. | Page 8 |
| Certainty of evidence | 22 | Present assessments of certainty (or confidence) in the body of evidence for each outcome assessed. | Page - |
| **DISCUSSION** | | |  |
| Discussion | 23a | Provide a general interpretation of the results in the context of other evidence. | Page 8, 9 |
|  | 23b | Discuss any limitations of the evidence included in the review. | Page 8, 9 |
|  | 23c | Discuss any limitations of the review processes used. | Page 8 |
|  | 23d | Discuss implications of the results for practice, policy, and future research. | Page 8, 9 |
| **OTHER INFORMATION** | | |  |
| Registration and protocol | 24a | Provide registration information for the review, including register name and registration number, or state that the review was not registered. | Page 4 |
|  | 24b | Indicate where the review protocol can be accessed, or state that a protocol was not prepared. | Page 4 |
|  | 24c | Describe and explain any amendments to information provided at registration or in the protocol. | Page 4, 5 |
| Support | 25 | Describe sources of financial or non-financial support for the review, and the role of the funders or sponsors in the review. | Page |
| Competing interests | 26 | Declare any competing interests of review authors. | Page |
| Availability of data, code and other materials | 27 | Report which of the following are publicly available and where they can be found: template data collection forms; data extracted from included studies; data used for all analyses; analytic code; any other materials used in the review. | None |

*From:*  Page MJ, McKenzie JE, Bossuyt PM, Boutron I, Hoffmann TC, Mulrow CD, et al. The PRISMA 2020 statement: an updated guideline for reporting systematic reviews. BMJ 2021;372:n71. doi: 10.1136/bmj.n71

For more information, visit: <http://www.prisma-statement.org/>
